# The Evolution of In Vitro Fertilization Practices in Saudi Arabia: Historical Perspectives and Future Directions

**DOI:** 10.7759/cureus.97000

**Published:** 2025-11-16

**Authors:** Salma Baghdadi, Amany Shaltout, Hani R Karrar, Rehab Salah A Alhendi, Mahmoud I Nouh, Faisal Albeedh, Amal A Alahmad, Yasmin ElHakeem, Yaser N AlBalawi, Dina M Hassan, Naila Aljahdali, Anas A Arab, Zahra M Almuayrifi, Wedyan S Alghamdi

**Affiliations:** 1 Obstetrics and Gynecology, Dr. Samir Abbas Hospital, Jeddah, SAU; 2 Pharmacy, Al-Azhar University, Cairo, EGY; 3 Pharmaceutical Care, Dr. Samir Abbas Hospital, Jeddah, SAU; 4 Obstetrics and Gynecology, Ministry of Health, Makkah, SAU; 5 Pharmacy, King Fahad Armed Forces Hospital, Jeddah, SAU; 6 Pediatrics, Dr. Samir Abbas Hospital, Jeddah, SAU; 7 Obstetrics and Gynecology, King Abdulaziz Hospital, Jeddah, SAU; 8 Obstetrics and Gynecology, Reproductive Endocrinology, Infertility, Dr. Samir Abbas Hospital, Jeddah, SAU; 9 Clinical and Chemical Pathology, Cairo University, Cairo, EGY; 10 Laboratory Medicine, Dr. Samir Abbas Hospital, Jeddah, SAU; 11 IVF Laboratory, Dr. Samir Abbas Hospital, Jeddah, SAU; 12 Pharmacy, Ministry of Health, Jeddah, SAU; 13 Clinical Nutrition, King Abdulaziz University, Jeddah, SAU

**Keywords:** gynecology, ivf, obstetrics, obstetrics and gynecology (ob-gyn), theraputic

## Abstract

In vitro fertilization (IVF) has revolutionized assisted reproductive technologies (ART), profoundly transforming infertility treatment since its inception. This procedure involves fertilizing an egg with sperm in a controlled laboratory environment, aiming to transfer the resulting embryo into the uterus for a successful pregnancy. While facilitating innovations like preimplantation genetic testing to avoid genetic abnormalities, it addresses a number of issues that contribute to infertility, such as endometriosis, male factor infertility, blocked fallopian tubes, and unexplained infertility. The IVF process consists of several key steps: ovarian stimulation, egg retrieval, fertilization, embryo culture, and embryo transfer. Ovarian stimulation involves administering fertility medications to promote the development of multiple eggs, increasing the chances of viable embryos. Egg retrieval is performed using a minimally invasive technique, followed by fertilization through conventional insemination or intracytoplasmic sperm injection (ICSI), which is particularly beneficial in male infertility cases. While IVF offers significant hope, it also carries risks, such as ovarian hyperstimulation syndrome, multiple pregnancies, and potential long-term health implications for mothers and babies. Success rates vary widely based on factors like age and clinic expertise, typically declining after age 40. Ethical considerations surrounding IVF include the moral status of embryos and equitable access to treatment, necessitating scrutiny as the commercialization of IVF can impose financial burdens on couples. This review will explore the evolution of IVF in Saudi Arabia, highlighting significant developments, challenges, and future research opportunities in this vital area of reproductive health. By addressing both technical and ethical dimensions, we aim to provide a comprehensive understanding of IVF's impact and potential in the region.

## Introduction and background

In vitro fertilization (IVF) represents a transformative advancement in assisted reproductive technologies (ART), significantly changing the landscape of infertility treatment since its introduction. IVF involves fertilizing an egg with sperm outside the body in a controlled laboratory environment, with the ultimate goal of transferring the resulting embryo into the uterus to achieve a successful pregnancy. This innovative procedure has offered hope to millions of individuals and couples facing infertility challenges globally, particularly since the birth of Louise Brown, the first baby conceived through IVF, in 1978 [1-4]. IVF is indicated for a variety of infertility factors, including blocked or damaged fallopian tubes, as well as male factor infertility (characterized by low sperm count, poor motility, or abnormal morphology), endometriosis, unexplained infertility, and genetic disorders [5-10]. It has also facilitated the advancement of preimplantation genetic testing, which is intended to prevent the transmission of genetic disorders. Consequently, IVF has become a widely accepted treatment option, with approximately 1.9% of all infants born each year as a result of IVF [11-14]. The IVF process typically involves several critical steps, including ovarian stimulation, egg retrieval, fertilization, embryo culture, and embryo transfer. During ovarian stimulation, fertility medications are administered to promote the development of multiple eggs, thereby increasing the likelihood of obtaining viable embryos for transfer. Egg retrieval is performed using a minimally invasive procedure, while fertilization can occur through conventional insemination or intracytoplasmic sperm injection (ICSI), particularly beneficial in cases of male factor infertility. After fertilization, embryos are cultured in the laboratory for a few days before being transferred to the uterus [15-17]. While IVF provides substantial hope for couples facing infertility, it is not without risks and complications. These can include ovarian hyperstimulation syndrome (OHSS), multiple pregnancies, ectopic pregnancy, miscarriage, and potential infections or bleeding [18-21].

Long-term health implications for both mothers and babies are also a consideration, highlighting the importance of careful monitoring and follow-up care. Success rates of IVF can vary widely based on several factors, including the woman's age, the underlying causes of infertility, and the expertise of the fertility clinic [22-23]. Generally, success rates decline with age, particularly after the age of 40. According to the Society for Assisted Reproductive Technology (SART), women under 35 have a live birth rate approaching 55%. Evaluating the success of an IVF cycle typically involves considering the endometrium (uterine lining), the embryo quality, and the embryo transfer procedure [24-26]. 

The practice of IVF also raises significant ethical considerations, including the moral status of embryos, the fate of unused embryos, the implications of preimplantation genetic testing, and equitable access to treatment. Ethical principles such as respect for autonomy, beneficence, non-maleficence, and justice play vital roles in guiding decision-making processes in IVF. Furthermore, the commercialization of IVF and associated financial pressures highlight the need for ethical scrutiny, as the costs of treatment can be prohibitive for many couples [27-30].

The IVF process involves several key steps aimed at achieving a successful pregnancy. First, the woman undergoes ovarian stimulation using fertility medications to encourage the development of multiple eggs, which enhances the likelihood of obtaining viable embryos for transfer or freezing [31-32]. Afterward, mature eggs are retrieved from the ovaries through a needle aspiration technique, usually performed under sedation and with ultrasound guidance. The retrieved eggs are then fertilized with sperm in a lab, either through conventional insemination, where sperm and eggs are mixed, or via ICSI, which involves injecting a single sperm directly into each egg, this method is particularly helpful in cases of male infertility [33-35]. Next, the fertilized eggs, known as zygotes, are cultured in the lab for 2 to 6 days to develop into embryos, with their growth closely monitored and graded for quality. Once developed, one or more embryos are transferred into the woman's uterus using a catheter. The decision on how many embryos to transfer is made thoughtfully to optimize the chances of pregnancy while minimizing the risk of multiples, with single embryo transfer (SET) being increasingly recommended, especially for younger women with high-quality embryos. Finally, to support implantation, the woman often receives progesterone after the embryo transfer to help maintain the uterine lining [33, 36, 37].

In summary, IVF has profoundly impacted the field of infertility treatment, providing hope and opportunities for countless individuals and couples. Ongoing advancements in technology and clinical practices have enhanced success rates and broadened the application of IVF. However, it is essential to address the ethical dimensions and potential risks associated with the procedure to ensure responsible and patient-centered care. As ART continues to develop, maintaining an open dialogue and ethical reflection will be crucial for navigating the complexities of IVF and maximizing its benefits for individuals and society. The future of IVF holds promise, with advancements in techniques such as cryopreservation and genetic screening potentially leading to even higher success rates and safer outcomes, affirming IVF's place as a cornerstone of modern reproductive health [17, 24, 28-30] . In this review article, we'll explore the journey of IVF in Saudi Arabia, looking back at its history and forward to what lies ahead. We'll share key developments, challenges, and advancements in this important field, giving you a thorough understanding of how IVF practices have evolved in the country. Our article will highlight the milestones that have significantly influenced the current state of assisted reproductive technologies in Saudi Arabia, while also considering exciting possibilities for future research and improvements.

## Review

Pioneering developments in assisted reproductive technology (1930s-1970s)

The story of mammalian IVF began in the 1930s in the USA, when Pincus stirred controversy by claiming he had created rabbit offspring from eggs fertilized in vitro (Enzmann and Pincus, 1934) [38,39]. Later, Rock and Miriam Menkin, his coworker, took things further by experimenting with both fertilized and unfertilized eggs taken from patients during surgery, announcing. In 1944, three human eggs were fertilized and then cleaved (Rock and Menkin, 1944) [38,40]. Chang and Austin found capacitation in 1951, the essential process that allows sperm to undergo necessary changes in the uterus before fertilizing an egg [38,39]. This led Austin to evaluate earlier claims of successful in vitro fertilization, establishing five criteria that hadn't been met in humans: the use of properly capacitated sperm, avoidance of aged eggs, confirmation that sperm had entered the egg, exclusion of parthenogenesis, and the birth of genetically related offspring [38-40]. In 1959, Chang provided solid proof of IVF success in mammals by fertilizing unfertilized eggs from a rabbit and giving the embryos to a different rabbit, which then birthed healthy young. Four years later, researchers successfully fertilized hamster eggs, but they couldn't grow (Chang and Yanagimachi, 1963, 1964) past the two-cell stage [38,41]. 1968 saw the mouse egg become fertilized in vitro and grown to the two-cell stage, with those cells being transferred to mice, resulting in a viable fetus different from the host mother genetically (Whittingham, 1968) [38,39].

During the 1950s and 1960s, a number of scientists, including Landrum Shettles from Columbia University, pursued the challenging goal of human IVF. Shettles claimed to replicate earlier techniques using human eggs, but he never published convincing evidence of success. In 1973, he attempted IVF and embryo transfer for a Florida couple, but when coworkers discovered this experiment, it was halted, resulting in a drawn-out court battle and bad press. In the midst of a heated public debate regarding IVF and embryo transfer (ET) and their potential use in humans, Robert Edwards, a reproductive biologist from Cambridge, formed a partnership in 1968 with gynecologist Patrick Steptoe, an advisor based in Jean Purdy, a nurse-technician in Oldham, Lancashire from Cambridge. Edwards had previously focused on egg maturation, driven by a desire to understand and potentially prevent chromosomal anomalies like Down syndrome, Klinefelter syndrome, and Turner syndrome were initially discovered genetically in 1959. During his work in Edinburgh in the 1950s, he studied the chromosomal behaviour of meiotic mouse eggs in response to hormonal signals for ovulation. His precise timing of this process was aided by research with his wife, Ruth Fowler, demonstrating that appropriate hormonal treatments could stimulate egg maturation in adult female mice, paving the way for induced ovulation in women a few years later [38, 41-43].

When Edwards moved to Edinburgh in 1958 to be employed at the NIMR Mill Hill, he unearthed prior research by Chang and Pincus, which indicated that a mouse egg's meiotic development was initiated only by releasing it from its follicle. This suggested it was the follicle's ability to restrict the egg, mediated by cAMP. If human eggs could similarly undergo spontaneous maturation upon release, it would open up the possibility of studying this otherwise hidden process. Consequently, Edwards devoted the next six years to trying to mature eggs from various species, including humans, in vitro after ovarian biopsies. This process was prolonged partly because the timing between the rise of luteinizing hormone (LH) and the egg's reentry into meiosis was not well understood; it actually takes about 36 hours in women, significantly longer than the 12 hours observed in more commonly studied animals like rats and mice. In 1965, after moving to the Physiology Department in Cambridge in 1963, Edwards published two papers detailing the meiotic maturation timelines for eggs in several species, including humans. In his Lancet paper, he outlined both the potential and challenges of his work with remarkable foresight, emphasizing his primary goal of studying and preventing genetic diseases rather than just alleviating infertility. He proved the feasibility of preimplantation genetic testing (PGT) in rabbit embryos a few years ago, well before it was achieved in humans. Additionally, collaborating with geneticist Alan Henderson, Edwards developed the "production line theory" of egg production to explain why older women have higher levels of maternal aneuploidy, showing that the earliest mouse eggs entering meiosis during fetal development were more stable and ovulated earlier in life compared to those that entered meiosis later [38, 44-46]. 

It was not until 1968, after meeting Patrick Steptoe, that Edwards became convinced that IVF could be a viable solution for infertility in many couples. This belief was not surprising, given that in the 1960s, knowledge about infertility's incidence, causes, and treatments was limited in the UK, with reproductive sciences largely focused on concerns about overpopulation. At that time, Edwards was primarily engaged in research aimed at inducing immunity to sperm as a potential contraceptive method, while Steptoe had long been interested in treating infertility. One of Steptoe's significant contributions was pioneering laparoscopic surgery in the UK, which allowed for a relatively non-invasive examination of the abdomen to identify the causes of infertility in women. When Edwards partnered with Steptoe, he hoped that Steptoe would help him in addressing the issue of capacitation of sperm, a problem Edwards had been facing for four years. It was believed that sperm from the male ejaculate needed exposure to the female genital tract to become capable of fertilizing an egg. Edwards came across a publication by Steptoe indicating he could recover sperm from the oviduct using laparoscopy, which could then be utilized for fertilizing eggs that Edwards had grown in a lab. Nevertheless, their initial collaboration did not involve this sperm recovery, as Barry Bavister's research revealed that sperm could reach full fertilization capacity without needing to be exposed to the female tract, simply by being subjected to a higher pH. Edwards found this to be applicable to human sperm as well. Although Steptoe's name was included in the nature article from 1969 that presented the initial widely recognized explanation of successful IVF in humans, he had not contributed many, if any, of the eggs or capacitated sperm used in the study [38, 45, 47-48]. 

At that time, emerging data indicated that while in vitro maturation of eggs could facilitate the chromosomal processes necessary for development, it did not lead to adequate ooplasmic maturation. Consequently, Edwards, Steptoe, and Purdy shifted their focus to developing laparoscopic techniques for retrieving nearly matured eggs from their follicles, thus circumventing this issue. Their efforts faced various unresolved technical challenges in their pursuit of successful IVF. Undeterred, Edwards combined his extensive understanding of oocyte maturation timing with Steptoe's innovative laparoscopic technique. This method allowed them to collect multiple oocytes from patients' intact ovaries under anesthesia by using gonadotropin injections. The laparoscope provided enhanced visualization of the abdominal cavity through cold light delivered via a flexible fiber optic tube. This setup enabled clear observation of the ovaries and the aspiration of eggs by puncturing the follicles with a thin needle. Preliminary attempts at laparoscopic oocyte recovery may have started as early as late 1968. In 1969, the focus was on refining the timing and methods for egg recovery following oocyte maturation. Initially, follicles were aspirated using syringes, but in September 1969, a new suction device was introduced, featuring a bypass valve for better control of suction, which resulted in clearer collection of follicular fluids. After testing various suction pressures, they determined an optimal pressure of no more than 12 cm Hg, as higher pressures could damage the oocytes. Establishing the right timing for laparoscopic egg collection proved to be a difficult yet essential aspect of achieving successful IVF [38, 47-49].

Breakthrough achievements and challenges in IVF

The primary objectives were to extract oocytes from their pre-ovulation follicles and to ensure that multiple pre-ovulatory oocytes were available for collection. To manage the control of ovulation and the menstrual cycle, follicle development, and oocyte maturation, they gave gonadotrophic hormones, particularly human menopausal gonadotrophin (HMG), to induce follicle development, then the last stage of follicular maturation, human chorionic gonadotrophin (HCG) comes next. HCG was given when urinary estrogen levels were sufficiently high (over 75 mg/day), with the timing optimized for laparoscopic recovery, typically in the early morning or late evening to accommodate volunteer staff. Starting in September 1969, they adopted a regimen of three HMG injections between days two and nine, followed by 5000 IU of HCG on days nine to 11 of the menstrual cycle for optimal results. The interval between the administration of HCG and egg collection was adjusted, with the timing compared to the presence of corpora lutea to verify whether ovulation had occurred before laparoscopy. Initially, laparoscopy was conducted on days 10-12, with the interval between HCG and the procedure starting between 28.75 and 29.50 hours, rising to between 29.50 and 30.00 hours by the middle of January 1970, and then reaching 32.0 and 33.5 hours by the middle of March 1970. In 1969, the average yield of eggs per follicle was only 33%, and many recovered eggs were found to be immature upon cytological or chromosomal examination. This prompted the team to cultivate the eggs for one to four hours in droplets of their own follicular fluid prior to insemination with capable sperm in a modified medium originally created by Barry Bavister for research on hamster fertilization, subsequently transitioning to a pH 7.55 Earle's-based medium. By 1970, around half of the follicles were yielding eggs, with the first attempts at fertilization occurring between October 23 and November 24, 1969. Edwards, Steptoe, and Purdy were able to effectively accomplish cleavage and blastocyst production in vitro between 1969 and 1971. In December of that year, they began transferring embryos (primarily between cells 8 and 16) to women in an effort to induce pregnancy. Notwithstanding the extensive record of scientific and technological advancements before them, along with their own breakthroughs, Edwards, Steptoe, and Purdy faced significant challenges in achieving successful implantation. Louise Brown was born in 1978, nearly ten years after the first human egg was successfully fertilized in vitro in 1969. They grappled with two main issues: first, they weren't sure if the embryos they were transferring had the capacity to grow further; second, they were uncertain whether the hormonal treatments required for stimulating multiple follicles might disrupt the conditions needed for implantation. On the bright side, their extensive experience with animal embryo culture provided some encouragement. By the 1950s, researchers had established that mouse embryos needed glucose to grow from the eight-cell stage to the blastocyst stage, and transferring these embryos into recipient uteri had resulted in live births. By the early 1970s, it became clear that two-cell mouse embryos could also be successfully cultured in vitro to the blastocyst stage and transferred to the uterus, resulting in live young. Some embryos could even go from the pronuclear stage to the blastocyst stage with successful outcomes [38, 50-53]. 

Additionally, it was established that culturing embryos needed pyruvate or lactate from the two-cell to the eight-cell stage. Brinster introduced a groundbreaking method in 1963 for growing mouse embryos under paraffin oil in tiny droplets of media, as opposed to using bigger tubes for testing, as previously the norm. Edwards adopted this approach starting in January 1970. Despite these advancements in culture conditions, it was evident that most embryos from various species tended to block development during in vitro culture at a stage specific to their species, which coincided with the crucial time when genes of the embryo become activated. This limitation had previously hindered the successful application of animal IVF. Moreover, early and untimely transfer of animal embryos to the uterus, before they would naturally reach, caused embryo mortality. If similar issues were present in humans, it would have complicated Edwards's work. Fortunately, no developmental block was observed in humans, and research by Marston and colleagues found that synchronization requirements were less strict in primates compared to rodents [38, 54-56]. 

This issue proved to be more challenging. Although they began transferring embryos in 1972, it wasn't until 1975 that they first confirmed a clinical pregnancy, which unfortunately turned out to be ectopic. The following year, they identified a pregnancy that was biochemical through a temporary increase in HCG blood levels. Edwards recognized that the elevated estrogen levels during the follicular stage, caused by the increased dosages of HMG, were detrimental to the endometrial luteal phase and actually reduced the luteal phase. Consequently, at the start of 1973, they attempted to lower estrogen levels by replacing HMG activation using cycles induced by clomiphene plus HCG to promote follicular development. But these cycles yielded extremely poor rates of egg recovery and were subsequently discontinued, leading to a return to HMG use from 1975 to 1977. To provide exogenous support for the luteal phase, they tried administering Pregnyl (gonadotrophinum chorionimum) starting in October 1972, and later combined it with intramuscular progesterone during 1973-1974. In July 1975, they added Primolut depot (hydroxyprogesterone hexanoate) to their regimen, combined with sporadic supplementation with Ritodrine, Indocid, and ethinyl estradiol [38, 57-59]. 

The introduction of daily bromocriptine from the mid-follicular phase through the luteal phase between February and July 1977 occurred when it was discovered that many patients had increased prolactin levels. Nevertheless, these efforts were unsuccessful, leading to the decision in late 1977 to eliminate the use of exogenous hormones to avoid their negative impact on estrogen levels and the luteal phase. This shift to natural cycles, along with more detailed endocrine monitoring, resulted in fewer eggs being recovered but improved the overall success in obtaining viable preovulatory oocytes for IVF, ultimately contributing to Louise Brown's birth. This achievement, along with a second birth in 1979 and two miscarriages, was accomplished by collecting the single egg that was naturally ovulated each cycle. The process involved regularly collecting urine samples every two to three hours to measure the rising levels of LH, which helped determine the optimal timing for laparoscopic egg recovery just before natural ovulation - a significant milestone [38, 51, 57, 60].

The expansion of IVF and ART practices globally

Edwards, Steptoe, and Purdy's achievements in 1978 and 1979 paved the way for the quick development of IVF and embryo transfer in clinical settings, leading to millions of IVF births worldwide. However, after Patrick Steptoe reached retirement age, the three pioneers had to relocate from Oldham. Unfortunately, this move lacked support from both the NHS and Cambridge University, leading to a significant pause until 1982, when they were looking for private facilities and established the required facilities at Cambridgeshire's Bourn Hall. In this period, the initiative shifted to Melbourne, Australia, where two research groups had been exploring IVF since the early 1970s, inspired by Edwards' successful IVF demonstration in 1969. In Melbourne, the first IVF births occurred, utilizing the natural cycle approach that Edwards and his colleagues had successfully implemented [38, 59-61]. 

Significant advancements were made in Melbourne, particularly in the use of clomiphene citrate to regulate ovulation hormonally, either with or without HCG, to recover eggs for IVF. This was soon followed by the successful use of HMG and HCG in the USA. The first instance of egg donation also took place, along with the successful freezing of embryos, which led to a pregnancy for the first time. However, this period of Australian leadership in IVF was interrupted in 1984 by the implementation of vague and constrictive laws in Victoria state, which stifled the previously innovative environment. Initially, IVF was a collaborative effort, allegedly sparked by discussions with Edwards in 1970 between Monash University-affiliated Queen Victoria Hospital's Carl Wood and John Leeton, and later by Ian Johnston and James Brown at the Royal Women's Hospital, linked to Melbourne University [38, 61-62]. 

In 1971, the team was strengthened by the addition of embryologist Alex Lopata, marking the serious start of their work. This accomplished Australian group reported their first two biochemical pregnancies from IVF in 1973, though neither progressed. Alan Trounson joined Wood's group at Monash University in 1977. A graduate of Sydney University, Trounson had completed post-doctoral training at the ARC Animal Research Station in Cambridge, where he gained experience in egg maturation, in vitro fertilization, and embryo transfer and freezing in cows and sheep, and where he had met Edwards. In 1979, the first ongoing pregnancy was reported at the Royal Women's Hospital in Melbourne, leading to the birth of the third verified test-tube baby in history, born in June 1980. But the publicity surrounding that hospital caused tension with the team of Monash, resulting in a division between the two clinics, with Melbourne's Johnston and Lopata and Monash's Wood and Trounson. This division had negative implications for legislative regulation, compounded by the initial widespread hostility from the Australian medical community and funding bodies [38, 62,63]. 

IVF in Saudi Arabia

The journey of IVF in Saudi Arabia began in the late 1980s. The first successful IVF procedure in the country was performed in 1986 at King Fahd Medical City in Riyadh. The birth of the first 'test-tube baby' in Saudi Arabia and the Middle East was a significant milestone, attributed to gynecologist Samir Abbas. This marked a significant milestone, as it made ART more accessible to Saudi couples who were struggling with infertility. A patient from Riyadh, who had struggled with infertility for eight years, successfully underwent treatment under his care. After becoming pregnant, she received prenatal care at the Military Hospital in Riyadh and ultimately gave birth to her son. This event marked the start of a new era in medical technology in Saudi Arabia and the Middle East. The first quadruplets conceived through laboratory fertilization in the Kingdom and the entire Arab and Islamic world were born under the supervision of Samir Abbas in 1988 to a Saudi patient from Medina. Although they were born prematurely at seven months, all four children are now healthy and have recently turned 24, with some now attending university. Their growth and intelligence levels are completely normal. The mother's infertility was attributed to an abnormality in the uterine lining. In 1992, the birth of quadruplets through laboratory fertilization, overseen by Samir Abbas, was recognized internationally as the third most significant case of its kind [64-65].

In 2006, Dr. Samir Abbas's medical center celebrated the birth of the first healthy twins born to parents with sickle cell anemia. This milestone was made possible by an innovative technique, the first of its kind in the Middle East, which allowed parents with hereditary blood disorders to have twins free from genetic conditions [65-67]. 

In the 1990s and early 2000s, reproductive medicine in Saudi Arabia experienced significant advancements. Numerous fertility clinics opened throughout Saudi Arabia, with notable centers located in Jeddah, Riyadh, and Dammam. These clinics provided various services, including IVF, intracytoplasmic sperm injection (ICSI), and fertility preservation methods. By the late 2000s, Saudi Arabia had established a strong infrastructure for IVF. The Saudi Society for Reproductive Medicine and the Saudi Fertility Society became key organizations dedicated to promoting research, education, and the exchange of best practices among professionals in the field [64,68].

IVF has truly changed the landscape of reproductive medicine, bringing hope to many couples struggling with infertility. In Saudi Arabia, a country rich in cultural and religious traditions, this technology has been embraced over the last few decades.

Ethical consideration of IVF in Saudi Arabia

The Saudi government has been instrumental in regulating IVF practices. In 2003, the Ministry of Health released guidelines that set standards for ART facilities, prioritizing patient safety and ethical compliance. These guidelines highlighted the necessity of informed consent and the responsible management of embryos. Ethical considerations are crucial in the context of IVF in Saudi Arabia. The influence of religious authorities continues to shape public views and acceptance of reproductive technologies [69-70].

Current trends and innovations

IVF practices in Saudi Arabia have become increasingly advanced. Innovations in genetic testing, such as preimplantation genetic testing (PGT), enable the screening of embryos for genetic disorders, enhancing the likelihood of successful pregnancies and healthy children. Additionally, the incorporation of modern technologies like artificial intelligence (AI) and telemedicine is reshaping the fertility treatment landscape. Remote consultations and virtual monitoring have made IVF more accessible, particularly for patients in rural areas. AI technology is revolutionizing infertility care at Dr. Samir Abbas Hospital, where it assists doctors with medical opinions based on the latest guidelines, generates structured medical notes from consultations, and performs data analysis on hospital data for better visualization of trends and patient outcomes. This integration enhances the quality of medical reports, ensuring accuracy and clarity while facilitating improved communication. Additionally, the IVF Virtual Training Program supports trainees, such as fellows, by providing easy access to relevant information and employs voice assistant technology to help patients navigate their healthcare journey, including booking appointments and addressing common queries. Overall, the implementation of AI at Dr. Samir Abbas Hospital improves the quality of care, streamlines operations, and supports ongoing medical education in the field of infertility [70-73].

## Conclusions

The evolution of IVF has profoundly transformed infertility treatment in Saudi Arabia, bringing hope to countless individuals and couples facing reproductive challenges. The integration of innovations such as preimplantation genetic testing and artificial intelligence has further improved the effectiveness and accessibility of IVF services, making them more patient-centred. However, ethical considerations remain crucial, as cultural and religious values significantly influence discussions surrounding reproductive technologies. As Saudi Arabia continues to advance in reproductive medicine, fostering ongoing dialogue about ethical practices and ensuring equitable access will be vital in realizing the full benefits of IVF for all seeking assistance. The future of IVF in the region is promising, paving the way for enhanced patient outcomes and a deeper understanding of reproductive health.
